# Editorial: Sleep disorders and airway diseases

**DOI:** 10.3389/fneur.2023.1264665

**Published:** 2023-08-18

**Authors:** Sy Duong-Quy, Naricha Chirakalwasan, Vinh Nguyen-Nhu, Timothy Craig

**Affiliations:** ^1^BioMedical Research and Sleep Lab Center, Lam Dong Medical College, Dalat, Vietnam; ^2^Immuno-Allergology Division, Hershey Medical Center, Penn State Medical College, Hershey, PA, United States; ^3^Excellence Center for Sleep Disorders, King Chulalongkorn Memorial Hospital, Bangkok, Thailand; ^4^Division of Pulmonary and Critical Care Medicine, Department of Medicine, Faculty of Medicine, Chulalongkorn University, Bangkok, Thailand; ^5^Department of Respiratory Functional Exploration, University Medical Center, University of Medicine and Pharmacy at Ho Chi Minh City, Ho Chi Minh City, Vietnam

**Keywords:** sleep disorder, airway disease, OSA, COPD, OLDOSA

Various medical disease states frequently exhibit sleep disorders, which have negative effects on patients' quality of life. Due to their detrimental effects on patients' neuropsychology and medical conditions, sleep disorders cause patients' comorbidities to become more severe and uncontrollable. Therefore, it is crucial to take a strategic approach to the diagnosis and treatment of sleep disorders in medical pathologies. Sleep disorders and airway diseases are closely related among these medical pathologies. Asthma and chronic obstructive pulmonary disease (COPD), as well as COVID-19 and post-COVID-19 diseases, can affect sleep architecture, lower the quality of sleep, and cause refractory insomnia or symptomatic obstructive sleep apnea (OSA). These reciprocal effects negatively impact the neuropsychological status, quality of life, and overall health of patients. Thus, the Research Topic “*Sleep disorders and airway diseases*” achieves great success with 15 published articles that cover all the aspects of sleep, from airway disease interactions in age-related populations and comorbidities to diagnosis and treatment approaches based on the use of technology and artificial intelligence (AI).

Firstly, OSA is the typical disease entity that manifests as a result of the interaction between sleep disorders and the upper airway at night. In children with adenotonsillar hypertrophy, the prevalence of OSA is high due to the occlusion of the upper airway. The use of respiratory polygraphy is more comfortable for children and is used to confirm the diagnosis of OSA. In this Research Topic, Tran-Minh et al. demonstrated a significant correlation between the apnea-hypopnea index (AHI) and the severity level of tonsillar and adenoid hypertrophy in children with snoring. Interestingly, treatment with antileukotriene receptors (ALRs) for non-severe hypertrophy or surgical therapy for those with severe hypertrophy could significantly reduce the mean AHI in this population with OSA. In fact, ALRs could be considered a first-line anti-inflammatory treatment for children with OSA and asthma. In addition, the cohort study conducted by Duong-Quy et al. consisting of 139 asthmatic children aged more than 5 years with comorbid OSA also showed that the severity of asthma and the symptoms related to OSA in these children significantly improved after 3-month-long and 6-month-long treatment with LRA combined with standard therapy for asthma. Moreover, asthma and OSA are the most common chronic respiratory disorders in children within bidirectional correlation. If left untreated, OSA may cause attention deficit hyperactivity disorder (ADHD) symptoms in asthmatic children. Therefore, prompt diagnosis of OSA will lead to an accurate control strategy in patients with asthma (Nguyen-Ngoc-Quynh et al.).

Recently, the overlap between obstructive lung disease (OLD) and OSA (OLDOSA) has been identified as a common phenotype of subjects with OSA. Therefore, personalized approaches to the diagnosis and treatment of subjects with OLDOSA are necessary in clinical practice. Besides OSA, the sleep quality of patients with asthma and COPD should be considered a crucial outcome in the management of these patients. The results of a cross-sectional study consisting of 390 patients with asthma and COPD conducted by Aldabayan and published in this Research Topic revealed that these patients had significantly reduced sleep quality, anxiety, and depression. The association between sleep duration, respiratory symptoms, asthma, and COPD in adults has been also well-demonstrated by Ruan et al.. The results of this study suggested that both long and short sleep duration might be associated with cough and dyspnea and that short sleep duration may be an independent risk factor for wheezing, asthma, and COPD. This relevant information might provide new insights into the management of respiratory symptoms and sleep quality.

Secondly, many subjects with acute respiratory symptoms due to COVID-19 suffer from post-COVID-19 sleep disorders. An online survey of the post COVID-19 conditions in various countries showed that nearly 80% of subjects had sleep disorders, including insomnia, sleep-disordered breathing, central disorders of hypersomnolence, circadian rhythm sleep-wake disorders, parasomnias, and sleep-related movement disorders (Tedjasukmana et al.). Thus, sleep disorders are the major problem in post-COVID-19 conditions and may affect patients' quality of life (QoL), making the existence of sleep disturbances a concern in the period post-COVID-19 (Tedjasukmana et al.). Furthermore, sleep disorders may also be seen in acute or chronic non-infectious diseases such as after a stroke or in the case of Down syndrome. This Research Topic also includes the results of the study on the relationship between sleep disorders and the prognosis of neurological function after stroke (Zhang et al.). In fact, low sleep quality and unusual nocturnal total sleep time (long or short) may be associated with short-term poor neurological function after stroke, and a high risk of OSA may be associated with a higher risk of all-cause death after stroke (Zhang et al.). A recent descriptive cross-sectional study conducted by Hoang-Anh et al. which was published in this Research Topic suggests that OSA may be a prognostic factor of cerebral infarction as well as cardiovascular diseases such as hypertension. Moreover, poor sleep quality is recognized as a major risk factor for poor health, increasing the incidence of serious chronic diseases. Additionally, the prevalence of OSA is significantly greater in patients with Down syndrome compared to the general population as a result of genetic, anatomical, endocrine, and metabolic abnormalities (Nguyen et al.). The consequences of sleep disruption due to OSA are very serious, especially in terms of neurocognitive and cardiovascular effects, leading to reduced life expectancy and quality of life in this population.

Thirdly, sleep medicine has reaped the benefits that have come with the progress of modern technology in terms of diagnosis and treatment by using machine learning, wearable devices, smartphone applications, and telemedicine. The primary results in this field have contributed to one-third of published manuscripts in this Research Topic. Liu et al. showed the effectiveness of using a machine learning approach for differentiating OSA patients with and without mild cognitive impairment and providing potential neuroimaging evidence for cognitive impairment caused by OSA. Particularly, during the COVID-19 pandemic, due to a shortage of medical staff and equipment, diagnosing sleep disorders and OSA became more difficult than ever before (Tran et al.). The state-of-the-art review published on this Research Topic suggests that the digital transformation of healthcare might provide the advantage of cutting-edge technologies and innovations that deliver sustainable service and medical solutions to patients, medical staff, and healthcare bodies (Tran et al.). The authors of this review also suggest that home sleep apnea tests could be a promising alternative sleep study solution for patients with OSA because it may help to save time and money while enabling improved interaction between physicians and patients. Polysomnography (PSG), which is manually scored by a sleep technologist, is currently the gold standard for diagnosis of OSA severity. However, PSG scoring takes time and effort and has a lot of inter-rater variability. Hence, PSG autoscoring can be done by using a deep learning-based software module for sleep analysis. Choo et al. have demonstrated the potential of PSG autoscoring in reducing the burden of manual scoring by sleep technologists, suggesting operational significance for sleep laboratories in the healthcare setting.

Finally, in this Research Topic, physical therapy through a smartphone application for home-based physical therapy for patients with OSA has been studied (Bui-Diem et al.). This application provides video and in-text tutorials for users to follow at home and a scheduler function to assist the user in organizing the training program, which may improve the efficacy of home-based physical therapy in patients with OSA. Within the same objective, Thai authors have used the Nitra application to study the effectiveness of the first internet-based cognitive behavioral therapy for insomnia (CBT-I) in their country (Theppornpitak et al.). The results of this interventional study confirmed the effectiveness of this first internet-based CBT-I by using the Nitra application for improving sleep efficiency and other sleep parameters in patients with chronic subthreshold to moderate insomnia. Finally, the role of the telemedicine management platform in the management of OSA should be considered. The results of the final study included in this Research Topic demonstrated that CPAP therapy may present distinct trajectories of adherence over time in addition to the traditional binary classification (Yi et al.). Interestingly, this study revealed that self-reported sleep health issues and psychological characteristics might be predictors of different adherence subtypes in patients with OSA. Thus, the authors stated that understanding CPAP use, profiles, and their predictors enable the identification of those who may require additional intervention to improve adherence and further enhance its therapeutic effect in OSA patients (Yi et al.).

In conclusion, this Research Topic gives an overview of the multidirectional correlation and interaction between sleep disorders and airway diseases. Especially, the application of advanced and revolutionary technologies in the diagnosis and treatment of OSA for personalized management of patients with airway diseases is necessary for the future. We hope that the readers of this Research Topic may benefit from useful information in the emerging field for their clinical practices.

## Author contributions

SD-Q: Conceptualization, Validation, Writing—original draft, Writing—review and editing. NC: Conceptualization, Supervision, Validation, Visualization, Writing—review and editing. VN-N: Conceptualization, Validation, Visualization, Writing—original draft, Writing—review and editing. TC: Conceptualization, Methodology, Validation, Visualization, Writing—review and editing.

